# “Dispatcher, Can You Help Me? A Woman Is Giving Birth”. A Pilot Study of Remote Video Assistance with Smart Glasses

**DOI:** 10.3390/s23010409

**Published:** 2022-12-30

**Authors:** Silvia Aranda-García, Myriam Santos-Folgar, Felipe Fernández-Méndez, Roberto Barcala-Furelos, Manuel Pardo Ríos, Encarna Hernández Sánchez, Lucía Varela-Varela, Silvia San Román-Mata, Antonio Rodríguez-Núñez

**Affiliations:** 1 GRAFAIS Research Group, Institut Nacional d’Educació Física de Catalunya (INEFC), Universitat de Barcelona, 08840 Barcelona, Spain; 2 CLINURSID Research Group, Psychiatry, Radiology, Public Health, Nursing and Medicine Department, Faculty of Nursing, University of Santiago de Compostela, 15782 Santiago de Compostela, Spain; 3 REMOSS Research Group, Faculty of Education and Sport Sciences, Universidade de Vigo, 36310 Pontevedra, Spain; 4 School of Nursing from Pontevedra, Universidade de Vigo, 36004 Pontevedra, Spain; 5 Department of Obstetrics, Complexo Hospitalario Universitario de Pontevedra, SERGAS, 36002 Pontevedra, Spain; 6 Faculty of Nursing, Catholic University of Murcia (UCAM), 061 Emergency Services (112) of Murcia, 30107 Murcia, Spain; 7 Faculty of Health Sciences of Melilla, University of Granada, 52005 Melilla, Spain; 8 Pediatric Critical, Intermediate and Palliative Care Section, University Clinical Hospital of Santiago de Compostela, 15706 Santiago de Compostela, Spain; 9 Primary Care Interventions to Prevent Maternal and Child Chronic Diseases of Perinatal and Developmental Origin (RICORS), RD21/0012/0025, Instituto de Salud Carlos III, 28220 Madrid, Spain; 10 SICRUS Research Group, Health Research Institute of Santiago de Compostela (IDIS), 15706 Santiago de Compostela, Spain

**Keywords:** technology, wearable, telemedicine, midwifery, natural childbirth, lifeguard, smart glasses, emergencies

## Abstract

Smart glasses (SG) could be a breakthrough in emergency situations, so the aim of this work was to assess the potential benefits of teleassistance with smart glasses (SG) from a midwife to a lifeguard in a simulated, unplanned, out-of-hospital birth (OHB). Thirty-eight lifeguards were randomized into SG and control (CG) groups. All participants were required to act in a simulated imminent childbirth with a maternal–fetal simulator (PROMPT Flex, Laerdal, Norway). The CG acted autonomously, while the SG group was video-assisted by a midwife through SG (Vuzix Blade, New York, NY, USA). The video assistance was based on the OHB protocol, speaking and receiving images on the SG. The performance time, compliance with the protocol steps, and perceived performance with the SG were evaluated. The midwife’s video assistance with SG allowed 35% of the SG participants to perform the complete OHB protocol. No CG participant was able to perform it (*p* = 0.005). All OHB protocol variables were significantly better in the SG group than in the CG (*p* < 0.05). Telemedicine through video assistance with SG is feasible so that a lifeguard with no knowledge of childbirth care can act according to the recommendations in a simulated, unplanned, uncomplicated OHB. Communication with the midwife by speaking and sending images to the SG is perceived as an important benefit to the performance.

## 1. Introduction

In developed countries, unplanned and unattended by midwives out-of-hospital birth (OHB) is a very rare event (0.07% in Spain) [1], which usually occurs because the pregnant woman is far from the health center or because the childbirth is triggered suddenly in multiparous women [2,3,4]. The worldwide prevalence of unplanned OHB is estimated to be 0.19–0.61% [5,6]. Of them, 35% occur without medical care and are attended by family members or bystanders (14% by a single person) [4]. Good care for the pregnant woman and the baby in an unplanned OHB can help the mother feel accompanied, have fewer injuries, and the baby to be healthier and prevent disabilities [4,7,8]. Although they are not alarming data, unplanned births are associated with significant perinatal morbidity and mortality [4,5].

Some environments, such as at the beach, with elevated temperatures in warm seasons, may increase the risk of abruption in women whose pregnancies are near or at term [9]. When childbirth occurs on a beach, the lifeguard will have to assist the pregnant woman until the arrival of medical emergency services. Lifeguards are emergency professionals with the duty to assist; they are experts in drowning and have basic knowledge of first aid [10], but they have hardly any knowledge of or experience in childbirth care. Therefore, similarly to other emergencies, as a layperson, he/she may be assisted by telephone through the emergency center [11,12], as has occurred in some OHB (12.5%) [4]. Ideally, the assistance should be carried out by a midwife, who is the health professional in charge of care for women of childbearing age and, specifically, care for physiological childbirth [13,14]. The midwife can assist the lifeguard based on the clinical practice guidelines for OHB that can be extended to first responders who are not experts in childbirth care, such as lifeguards or firefighters [15].

Advances in communication technologies could favor assistance in this type of out-of-hospital emergency by allowing the teleoperator to visualize the situation in real time and communicate with the person providing guidance in the procedures to be carried out [16]. However, remote assistance using smartphones has certain limitations that advise against its use, especially when there is only one intervener, who must manipulate the device at the same time as performing the assistance [11,12,17].

Smart glasses (SG) could be a breakthrough in these scenarios, allowing hands-free communication in real time and the transmission of images and videos from the teleoperator to the layperson [16,18,19]. In this type of telemedicine, defined as “the transfer of skills between health professionals telematically” [20], wearable glasses allow the wearer to read and/or see information (e.g., pictures, videos, checklist, etc.) in the lens of the glasses. The professional can also receive audio communication and complete video calls, which further distinguishes SG as a new device with potentially significant advantages over other devices previously used in telemedicine. To date, very few studies have analyzed the usefulness of this technology in the out-of-hospital setting [21,22], and none have focused on childbirth care or the role of the midwife in telecare.

With the hypothesis that interactive guidance by a midwife visualizing in real time what a layperson transmits to him/her through SG will allow this person to perform the appropriate procedures in a simulated, uncomplicated childbirth, we conducted the present study, whose main goal was to evaluate the possible advantages of this aid.

## 2. Materials and Methods

### 2.1. Design

Quasi-experimental randomized pilot study in a simulated scenario.

### 2.2. Participants

**Figure 1:**
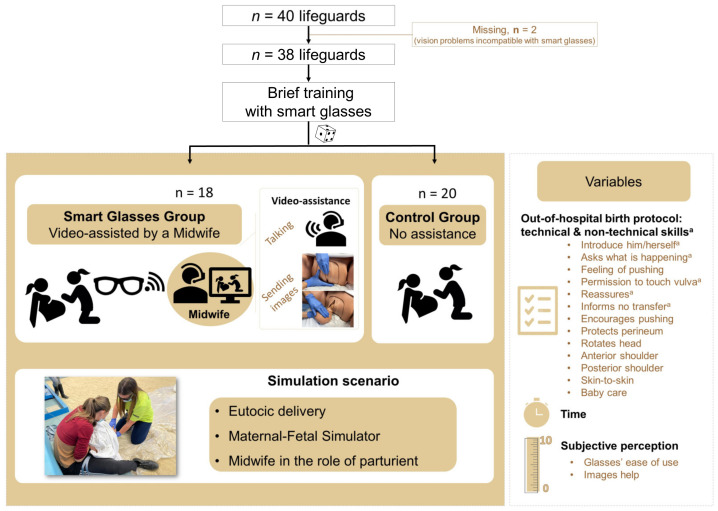
Flowchart design and sample of participants.

Lifeguards who had studied aquatic lifesaving at a university of physical activity and sports sciences were invited to participate. As inclusion criteria, they must be without theoretical or practical training related to childbirth, and without personal birth experience. Participants with visual impairments incompatible with the use of smart glasses were excluded. Ultimately, 38 lifeguards (9 women, 29 men) aged 22 ± 4 years old, with weight 51–102 kg and height 1.55–1.92 m, were included (Figure 1).

### 2.3. Brief Training on Smart Glasses and Connection Details

Each participant received a brief training with SG to become familiar with this device (Vuzix Blade Upgraded, Vuzix, New York, NY, USA). To ensure adequate familiarization with SG, all participants had to report a perception of the domain in their video call function of 3 or more on a 5-point Likert scale (5: perfect domain). The training was conducted on a simulated beach inside a university building with good internet connection. The SGs were connected via a 4G wireless network to make the video call with the VRA Mobile App (Vuzix, New York, NY, USA).

### 2.4. Clinical Simulation and Variables

Participants were randomized into the Smart Glasses group (SG: intervention) and the Control Group (CG). All participants had to perform in the same simulation of an imminent eutocic birth away from the hospital. All lifeguards wore smart glasses to record their performance. In addition, the SG group was video-assisted by an expert midwife. CG participants performed without external assistance (Figure 1).

Scenario: An instructor presented the clinical simulation: “A pregnant woman goes into labor on the beach. It is an imminent labor. There is a woman sitting on the ground with a maternal–fetal simulator”. For the SG group, the instructor also said, “You wear the smart glasses, which are connected to a midwife at the control center”, while for the GC group, he said, “You should try to help the laboring woman and the baby with the knowledge you have”. The researcher who handled the maternal–fetal simulator (PROMPT Flex, Laerdal, Norway) was an expert midwife (see Video S1 in Supplementary Material).

The midwife guiding the SG participants through the smart glasses was adapting the assistance based on what she observed and heard from the video call that provided streaming video through the front camera of the glasses. In addition, the midwife projected two images during the third stage of labor that each lifeguard could observe on their smart glasses: one about the placement of the hands for perineum protection and the other showing the anterior shoulder extraction aid (Figure 1).

The following variables were analyzed: (A) total action time (sec); (B) eutocic birth performance protocol measured dichotomously (performed/not performed) and categorizing the variables into technical or non-technical skills (see Figure 1 and Table 1); and (C) subjective perception variables were measured on a scale of 0–10 (ease of use of the smart glasses, with 10 being very difficult [23]), and opinion on whether the photos projected on the smart glasses facilitated the performance (with 10 being very high). The variables of the performance protocol are those stipulated by the clinical practice guideline for OHB [15] (see Table 1).

### 2.5. Data Analysis

Variables were expressed as mean (standard deviation), absolute, or relative frequencies (95% confidence intervals) as appropriate. The Shapiro–Wilk test was used to check the normal distribution of the continuous variables. Paired samples *t*-test or Wilcoxon Signed Rank test were used to compare continuous variables (*t*-test for parametric variables). The chi square test was used to compare dichotomous variables (those variables categorized as performed/not performed). Statistical analyses were performed using SPSS for Windows (v.21.0). Significance was established when *p* < 0.05.

## 3. Results

No CG participant was able to correctly perform the entire sequence of OHB. However, 35% of the SG participants managed to correctly perform the entire sequence (*p* = 0.005). All variables of the protocol, both technical and non-technical skills, were significantly better in the SG group than in the CG (*p* < 0.05) (Table 1). More than 90% of the SG participants correctly performed 11 of the 13 OHB performance variables. The other two variables were identifying oneself, which was performed by 75%, and placing the newborn skin-to-skin, which was performed by 80%. From the 14 variables of the childbirth assistance protocol, there were 8 that no CG participant (or only one participant) was able to perform (introduce him/herself, ask what is happening, ask about the sensation of pushing, ask permission to touch the vulva, inform that the transfer cannot be performed, actively protect the perineum, place the baby skin-to-skin, and dry and keep the baby warm).

Sixty percent of SG participants correctly performed all technical skills versus 0% of the CG (*p* < 0.001), and 55% of SG participants correctly performed all non-technical skills versus 6% of the CG (*p* < 0.001) (Table 2).

The performance time of all simulations was less than 5 min (CG: 117 ± 32 s, SG: 178 ± 37 s, *p* = 0.03). The SG participants reported that the difficulty of using the glasses was low (1.2 out of 10) and that the photos projected through the smart glasses made the performance much easier (8.6 out of 10) (Table 3).

## 4. Discussion

The potential of telemedicine is continually expanding, although additional evidence is needed before its incorporation into routine practice [24,25]. The scarce scientific evidence on the use of SG-dispatcher in out-of-hospital emergencies is limited to the field of triage [21,22]. In this sense, our study, whose aim was to assess the impact of the use of SG on the quality of care for a specific acute out-of-hospital event, in our case an uncomplicated birth, provides novel and relevant information.

Our major finding was that when the midwife video-assisted with SG, the lay rescuer in childbirth assistance is able to correctly perform the protocol for unplanned, uncomplicated OHB. When a birth occurs in an unplanned way, a process that should be natural can become an emergency with relevant risks for the mother and the baby [4]. Although rare, it is a reality that unplanned OHBs do occur, and providing a means that may help bystanders to perform better immediate care might improve mother and baby outcomes. Based on our findings, video assistance with SG guided by a midwife could be good assistance for laypeople naïve in this type of event, even if they have some duty to assist (e.g., lifeguards, police, firefighters, etc.).

It has been verified that with the video assistance, the participants were able to correctly follow the sequence of actions and perform the different recommended steps even though it was the first time they had done so. Improvements in video-assisted performance with SG occurred in both technical and non-technical skills. The technical skills are those directly related to procedural birth assistance. Without the help of a childbirth professional expert in these skills, it would be difficult to provide good care for both mother and baby. Non-technical skills are those related to communication and human care, in which the help of the midwife is not indispensable but is desirable, as these skills are essential in the management of this stressful situation.

Regarding technical skills, the video-assisted lifeguards with SG performed significantly better than the CG. This includes aspects related to the assessment of the stage of labor, assistance during the expulsive phase, and newborn care.

When the midwife at the emergency center realized that this was an unplanned OHB situation, he/she guided the lifeguard to ask about the pushing feeling, assess the stage of labor, encourage pushing in the third stage of labor, and protect the perineum. Many vaginal deliveries are accompanied by perineal trauma, which is associated with significant short- and long-term morbidity with pain, urinary or fecal incontinence, and dyspareunia [26,27,28]. Active protection of the perineum is an extended practice to reduce perineal injury [29] recommended by the World Health Organization [30] and current protocols [31,32]. In our study, the midwife’s SG-dispatcher allowed 90% of the participants with SG to correctly protect the perineum compared to 0% of those who acted autonomously.

Once the third stage of labor has ended, newborn care is essential. In the proposed setting, hypothermia used to be the most common morbidity, occurring in 50% of unplanned OHBs [33]. The wet newborn can lose temperature rapidly by evaporation and conduction [34]. Therefore, a good strategy to prevent hypothermia is to place the baby skin-to-skin on the mother’s bare chest and/or abdomen, then dry and cover him/her [34,35]. It is also advisable to place the baby skin-to-skin for additional benefits (successful breastfeeding, cardiorespiratory stability, and maternal–filial bonding) [36]. In our study, the vast majority (80%) of participants with SG placed him/her skin-to-skin and almost all dried and covered the baby’s body and head. However, none of the CG participants placed the newborn skin-to-skin, and only one dried and covered the baby.

Regarding nontechnical skills, the lifeguards without assistance with SG did not have effective communication with the pregnant woman: they did not speak to the pregnant woman to identify themselves, nor explain the situation, nor did they ask for permission to touch the vulva, and only half of them reassured the woman. However, with the SGs, the midwife was able to video-assist the rescuers, and nearly all of them performed the non-technical skills perfectly. Although in unplanned OHB the woman may feel confident, empowered, and exhilarated, communication and reassurance are especially important because expectant mothers report fear, and worry about poor interpersonal skills of the people assisting them [7,37]. This, added to the fact that pregnant women often report lack of consent for certain procedures such as vulvar assessment or active protection of the perineum [7,37], has led scientific societies to urge effective and empathic communication with the women [38,39]. Care providers should be aware of the importance of tone, behaviors, and the words they use for effective communication, ref [38,39] as was the case in the first responders with SG thanks to the video assistance of the midwife who encouraged better accompaniment of the woman in this simulated vital moment. These non-technical skills are fundamental for the respectful accompaniment of the woman so that the experience of childbirth can be positive and empowering instead of negative and traumatizing [40,41].

The positive results of the intervention group throughout the protocol can be explained by the good communication between the rescuer and the midwife through the SG. The midwife was able to guide and correct the rescuer based on what she saw/heard in the video call. This communication with SG helped the vast majority of SG participants to perform most of the required skills well. In addition to verbal instructions, the midwife also sent images so that the rescuers could see how they should assist in the third stage of labor (placing their hands to protect the perineum or to facilitate the extraction of the baby’s shoulders). These images were projected onto the lens of the SG themselves, and participants stated that they were very helpful (8.6 ± 2.1/10) to facilitate understanding how they should position their hands to assist the pregnant woman. In addition, participants stated that the use of the smart glasses’ technology was of very low difficulty (1.2/10).

Without the video assistance, the lifeguards, who were laypeople in childbirth care, could barely help in unplanned, uncomplicated OHB. Some of the participants were able to perform isolated actions that could be considered innate or transversal to any emergency situation, such as reassuring the pregnant woman, or accompanying in certain aspects of the expulsive phase instinctively, such as holding the baby’s head and shoulders in the expulsive phase.

In our pilot study, we have verified how telemedicine with images through SG can be a powerful communication device in which a midwife can remotely video-assist a lay person in an eutocic birth. In the field of unplanned OHB, this becomes even more interesting when they are assisted by a single person, which represents 14% of these deliveries [1], because SG allow hands-free, remote assistance. Based on our results, it could be interesting to study the incorporation of this type of new communication technology, especially in those places where delayed times are expected in the arrival of emergency services (for example: remote places such as rural areas, fishing boats, or airplanes, far from the ambulance and hospital). Although the future implementation of this technology may represent a great opportunity to improve assistance in emergencies attended by laypeople, it will also represent a challenge in its implementation. It will be necessary to take into account the opinion of the dispatcher, the first responder, and the patient (in this case, the pregnant woman) for the acceptability of this technology. In addition, relevant ethical aspects related to privacy, security, equity, and responsibility must be taken into account to respect the rights of people [42].

This study has some limitations. The emergency was simulated, which entails its specific limitations by design. Although the simulated pregnant woman was a midwife manipulating a maternal–fetal simulator in an attempt to make it as realistic as possible, these results cannot be directly extrapolated to a real situation. The use of this type of telemedicine requires good connectivity, which may limit its use in remote locations or in some areas of buildings with poor internet connection (e.g., basements, etc.). The type of birth chosen was eutocic, in which there were no complications for the pregnant woman or the baby, and we do not know the extent of the usefulness of this type of midwife-assisted telemedicine in other types of deliveries. Our findings are contextualized with a specific typology of participants (lifeguard workers, young people, university students, laypeople in childbirth assistance), so they cannot be extrapolated to other profiles such as other health professionals or people with a low proficiency with communication technologies. Also, our study did not compare classic telephone vs. smart glasses assistance in childbirth, an investigation that should be done in the future.

## 5. Conclusions

In simulated, unplanned, uncomplicated, remote birth conditions, telemedicine through video assistance with SG is feasible for a lifeguard with no knowledge of childbirth care to perform according to the recommendations for unplanned, out-of-hospital birth. The communication of the midwife with the lay attendant by talking and sending images to the SG may be of significant benefit both in relation to technical (steps of the recommended sequence) and non-technical skills (support to the laboring woman). The SGs are easy to use, with a minimal learning period, although they require good internet connection, which may be a limitation in remote locations or those with poor coverage. More studies are needed to ascertain the contribution of SG to telemedicine in out-of-hospital emergency care.

## Figures and Tables

**Table 1:** Unplanned out-of-hospital birth sequence, classification into technical or non-technical skills, and description of the variables.

Unplanned Out-of-Hospital Birth Sequence	Technical Skill	Non-Technical Skill	Skills Description
(1) Introduce him/herself		x	The lifeguard (L) introduces him/herself to the pregnant woman (P)
(2) Asks what is happening		x	L asks what is happening and how the P is
(3) Feeling of pushing	x		L asks the P if she has a feeling of pushing
(4) Permission to touch vulva		x	L asks the P for permission to examine her vulva
(5) Reassures		x	L reassures the P
(6) Informs no transfer		x	L informs P that childbirth is imminent and she cannot be transferred to a health center
(7) Encourages pushing	x		Expulsive phase (EP): L encourages the P woman to push
(8) Protects perineum	x		EP: L protects the P’s perineum with his hands
(9) Rotates head	x		EP: L enables the baby’s head to turn
(10) Anterior shoulder	x		EP: L holds the baby to enable the baby’s anterior shoulder to come out
(11) Posterior shoulder	x		EP: L holds the baby to enable the baby’s posterior shoulder to come out
(12) Skin-to-skin	x		L places the baby skin-to-skin in contact with its mother
(13) Baby care	x		L dries, wraps up, and warms the baby’s head and body

**Table 2:** Participants in the smart glasses video assistance group and control group who correctly performed the different techniques of the out-of-hospital birth sequence.

Variable	Description	Smart Glasses	Control	*p* ^b^
		*n* (%)	*n* (%)	
(1) Introduce him/herself ^a^	The lifeguard (L) introduces him/herself to the pregnant woman (P)	15 (75)	0 (0)	<0.001
(2) Asks what is happening ^a^	L asks what is happening and how the P is	19 (95)	2 (11)	<0.001
(3) Feeling of pushing	L asks the P if she has a feeling of pushing	19 (95)	1 (6)	<0.001
(4) Permission to touch vulva ^a^	L asks the P for permission to examine her vulva	18 (90)	1 (6)	<0.001
(5) Reassures ^a^	L reassures the P	18 (90)	9 (50)	0.007
(6) Informs no transfer ^a^	L informs P that childbirth is imminent and she cannot be transferred to a health center	18 (90)	0 (0)	<0.001
(7) Encourages pushing	Expulsive phase (EP): L encourages the P woman to push	19 (95)	11 (61)	0.011
(8) Protects perineum	EP: L protects the P’s perineum with his hands	18 (90)	0 (0)	<0.001
(9) Rotates head	EP: L enables the baby’s head to turn	20 (100)	8 (44)	<0.001
(10) Anterior shoulder	EP: L holds the baby to enable the baby’s anterior shoulder to come out	19 (95)	12 (67)	0.024
(11) Posterior shoulder	EP: L holds the baby to enable the baby’s posterior shoulder to come out	20 (100)	11 (65)	0.004
(12) Skin-to-skin	L places the baby skin-to-skin in contact with its mother	16 (80)	0 (0)	<0.001
(13) Baby care	L dries, wraps up, and warms the baby’s head and body	19 (95)	2 (11)	<0.001
Complete sequence		7 (35)	0 (0)	0.005
All technical skills		12 (60)	0 (0)	<0.001
All non-technical skills ^a^		11 (55)	1 (6)	<0.001

^a^ non-technical skill, ^b^ significance according to chi-square.

**Table 3:** Perceptual variables of competence, confidence, difficulty using the smart glasses, and image reception.

	Smart Glasses	Control	*p*
	n (%)	n (%)	
Self-reported level of competence	6.8 ± 1.5	3.8 ± 1.8	<0.001
Self-reported level of confidence	7.0 ± 1.4	3.3 ± 2.9	<0.001
Difficulty using smart glasses	1.2 ± 2.0	-	-
Images facilitate the help	8.6 ± 2.1	-	-

## Data Availability

Not applicable.

## Supplementary Materials

The following supporting information can be downloaded at: https://www.mdpi.com/article/10.3390/s23010409/s1, Video S1: Lifeguard assisting an imminent childbirth with the help of a midwife through smart glasses.
